# Oviposition Behaviors and Ontogenetic Embryonic Characteristics of the Western Tarnished Plant Bug, *Lygus hesperus*

**DOI:** 10.1673/031.012.3601

**Published:** 2012-03-14

**Authors:** W. Rodney Cooper, Dale W. Spurgeon

**Affiliations:** USDA-ARS, Western Integrated Cropping Systems Research Unit, 17053 North Shatter Ave, Shatter, CA 93263

**Keywords:** Hemiptera, Miridae, saliva, stylet probe

## Abstract

The western tarnished plant bug, *Lygus hesperus* Knight (Hemiptera: Miridae) is a key pest of fruit, vegetable, and field crops in the western United States, but many aspects of *L. hesperus* ecology are poorly documented. A sound understanding of oviposition behavior and characterization of the phases of embryonic development would be useful in studies of interactions between *L. hesperus* and its hosts, and in efforts to better understand the developmental consequences of low temperatures. Because *L. hesperus* insert their eggs into the host, most of the egg is obscured from view, and some aspects of oviposition and subsequent egg development cannot be observed directly. A novel observational method which took advantage of the propensity for *L. hesperus* to oviposit in semi-transparent sheets of agarose was used to observe oviposition and subsequent embryonic development. *Lygus hesperus* females stylet-probed prospective oviposition sites and during oviposition the ovipositor followed the path of the final probe. Oviposition, from insertion to withdrawal of the ovipositor, required ∼30 seconds. Identifiable phases of embryo development included egg swelling, katatrepsis, appearance of body segments and appendages, development of red pigmentation in the eyes and terminal antennal segments, and formation of the 3^rd^ embryonic cuticle. These phases were observed at about 0.3, 0.4, 0.5, 0.6, and 0.8 of the total duration between oviposition and hatch, respectively. Infertile eggs did not exhibit any of these phases. Our descriptions of embryonic development will facilitate the study of *L. hesperus* egg biology and ecology, and permit estimates of egg population age structure and prediction of egg hatch.

## Introduction

The western tarnished plant bug, *Lygus hesperus* Knight (Hemiptera: Miridae) is a key agricultural pest in the western United States that causes economic damage to a wide range of fruits, vegetables, forages, and field crops (Wheeler 2001). Current approaches for managing *L. hesperus* populations rely primarily on use of conventional chemical controls in conjunction with nominal treatment thresholds (Spurgeon and Cooper 2011). Development of ecologically-based management strategies, including components to address dispersal and landscape-level cropping patterns (Carrière et al. 2006) or use of biological controls against overwintering *Lygus* populations (McGuire et al. 2006) will require an accurate understanding of *L. hesperus* overwintering ecology.

*Lygus hesperus* enters a state of adult diapause in response to short photophases of late-summer and early-fall (Beards and Strong 1966; Leigh 1966; Strong et al. 1970). Although Spurgeon and Brent (2010) questioned the validity of the criteria used to distinguish diapause in earlier studies, the consensus has been that diapause in the California Central Valley terminates in latefall (Beards and Strong 1966; Leigh 1966) to early-winter (Strong et al. 1970). Upon diapause termination, adult *L. hesperus* mate and oviposit. However, reports of the timing of oviposition and fate of these eggs are variable. Beards and Strong (1966) reported that most *L. hesperus* adults die by midFebruary, and the population survives the winter as eggs which begin to hatch in late-March. Leigh (1966) reported that populations of adults could be low from mid-January to mid-March and that nymphs were common after mid-February. Based on an estimated lower temperature threshold for egg development of > 10 °C (Champlain and Butler 1967), Strong et al. (1970) deduced that eggs laid before 10 February would die and that nymphs appearing in April resulted from eggs laid after 10 February. Based on the report of Strong et al. (1970), the overwintering population of *L. hesperus* may represent a bottleneck vulnerable to manipulation if the dynamics of diapause induction and termination, as well as the rates and success of egg development at low temperatures, were better known.

Our preliminary studies of *L. hesperus* egg development in low temperatures were inconclusive, because it was not possible to determine definitively whether failure of egg hatch was caused by lack of development or by deterioration of green bean pods (*Phaseolus vulgaris* L.) used for oviposition substrate. The eggs of *Lygus* spp. and many other mirids are oviposited into plant tissues so that only the operculum is visible (Stewart and Gaylor 1993; Wheeler 2001; Ma et al. 2002). The positioning of *L. hesperus* eggs within typically opaque plant tissues prevents the continuous and non-destructive observation of oviposition and subsequent embryo development. In addition, Cooper and Spurgeon (2011) reported that *L. hesperus* females typically stylet-probe the oviposition substrate (green bean pods or cotton flower buds, *Gossypium hirsutum* L.), presumably at the site of intended oviposition. Better understanding of this behavior may provide insights into the selection of *Lygus*-tolerant cotton cultivars if such stylet-probing reflects an obligatory oviposition behavior and the mechanisms involved can be determined. These behaviors (stylet-probing and oviposition) are also effectively hidden from view by the opacity of natural oviposition substrates.

In preliminary experiments we determined that *L. hesperus* females would oviposit in semi-transparent sheets of agarose and that the agarose was less subject to deterioration than were natural oviposition substrates. Use of the agarose substrate facilitates detailed documentation of oviposition behaviors and phases of embryonic development that are important to the meaningful evaluation of basic *L. hesperus* biology and ecology. Knowledge of the developmental changes exhibited by *L. hesperus* eggs will also facilitate the estimation of age structures of populations of *L. hesperus* eggs. Therefore, our objective was to observe and document the oviposition and development of *L. hesperus* eggs within an agarose oviposition substrate.

## Materials and Methods

### *Lygus hesperus* colony

Newly eclosed *L. hesperus* adults were collected from a colony maintained on green bean pods and raw sunflower seeds (*Helianthus annuus* L.). Insects used in the study were ≤ 3 generations removed from field populations. Mated and gravid females were obtained by holding groups of 250 same-aged mixed-gender adults in 3.8 L buckets with a screened lid. Females were identified as mated by observing the presence of a spermatophore visible through their ventral abdominal walls. These mated females were used for observations of oviposition behavior and subsequent egg development. In addition, groups of 130 females were held in separate buckets to provide reproductively mature adults that were not mated. Previous observations of *L. lineolaris* egg development indicated eggs absorb water and expand in size (Stewart and Gaylor 1993), but it is not known whether water absorption is passive or requires a viable embryo. Therefore, eggs from unmated females were observed for comparison with egg development by fertile eggs. The buckets contained shredded paper and green bean pods and were held within an environmental chamber (Percival Scientific, www.percival-scientific.com) maintained at 26.6 ± 0.5 °C with 20% RH and 14:10 L:D photoperiod. Insects were used for experiments nine days after adult eclosion to ensure adults were reproductively mature (Strong et al. 1970).

### Observations of oviposition behavior

The semi-transparent oviposition substrate was prepared by first pouring a solution of 1.5% agarose (Sigma-Aldrich, www.sigmaaldrich.com) into a 50 mm diameter Petri dish (Microtech Scientific, www.microtechscientific.com) to a depth of 3–4 mm. After the agarose cooled, sections were cut with a razor blade and removed to leave a 10 × 50 mm strip of agarose bisecting the Petri dish. A single mated *L. hesperus* female was released into the Petri dish, and oviposition events were recorded for 4 hours using two high-definition Sony Handycam HDR-CX100 digital cameras (Sony Corporation, www.sony.com) at frame rates of 30/sec. One camera was positioned in the plane of the agarose strip and perpendicular to its long axis, whereas the second camera was positioned above and perpendicular to the upper surface of the strip. An incandescent lamp and two small fluorescent lamps positioned above and around the upper camera provided light. Cameras, lights, and the experimental arena were cloaked with a white cloth to minimize disturbance to the insect from activities within the laboratory. A small fan was used to ventilate the experimental arena, and temperature near the Petri dish was monitored with a HOBO Data Logger (Onset, www.onsetcomp.com). The temperature near the plate was typically ∼29 °C. Recordings were viewed using Adobe Premiere Pro CS4 (Adobe Systems, www.adobe.com). Fifteen females were recorded, but most did not oviposit in the agarose sheets within the 4hour recording time. Three mated females produced a total of 15 successful oviposition events and 12 unsuccessful attempts.

Oviposition in green bean pods was observed for comparison with oviposition in the agarose substrate. Green beans were cut into 2 cm long sections, and both ends of each section were sealed with melted paraffin. To allow an unobstructed view of oviposition, each green bean section was partially embedded horizontally in paraffin within a 50 mm Petri dish so that only the upper half of each section was exposed to *L. hesperus* females. Individual mated *L. hesperus* females released into the experimental arena were recorded for two hours using two high definition digital cameras. The cameras were positioned on opposite sides and above the bean section, perpendicular to its long axis and at an angle ∼45° from horizontal. A total of 23 successful oviposition events and 33 unsuccessful oviposition events were recorded from five different females.

### Observations of embryo development

Oviposition chambers were constructed from two 12-well tissue-culture dishes (BD Biosciences, www.bd.com, product number 352043). The two dishes were stacked, with the upper dish inverted over the lower dish so that the wells of the two plates were adjoining. Each well of the bottom dish was filled with 8 mL 1.5% agarose. After cooling, the agarosefilled wells were covered with stretched Parafilm to provide footing for ovipositing females. The upper dish was used to confine a mated or an unmated *L. hesperus* female to each agarose filled well. A 0.3 cm diameter ventilation hole was drilled in each well of the upper dish to minimize condensation within the chambers. A total of 48 females (24 mated, 24 unmated) representing two different cohorts of adults (12/mating state/cohort) were confined in the chambers for oviposition.

The oviposition chambers containing *L. hesperus* females were held in an environmental chamber maintained at 26.6 ± 0.5 °C with 20% RH and 14:10 L:D photoperiod. After ∼6 hours, the adults were removed, and the agarose in each chamber was searched for eggs. The position of each egg was noted, and the Parafilm cover was removed from the bottom dish to facilitate the unimpeded observation of embryonic development. During the period of egg development, the dishes containing eggs were maintained under the same environmental conditions as were used for the oviposition periods.

Changes in egg morphology and embryo development were monitored for 15 consecutive days or until eggs hatched (*n* = 28 eggs from mated females and *n* = 25 eggs from unmated females). Each egg was photographed near the same time each day at a magnification of 45 × using a digital camera mounted on a dissecting microscope (either a model DP 12 camera on a model S261 microscope, Olympus America, www.olympusamerica.com, or a model 11.2 Color Mosaic camera, Diagnostic Instruments, www.spotimaging.com, mounted on a model MZ125 microscope, Leica Microsystems, www.leica-microsystems.com). On each day, each egg was recorded by a series of photographs taken at different focal lengths. Depth of field was maximized by constructing a montage from each series of pictures using Helicon Focus software (Helicon Soft, www.heliconsoft.com). Characteristics associated with egg and embryo development were then recorded from the compiled photographs. The timing of appearance of the various phases of development was characterized as a proportion of the total egg development time of each egg. These proportions were calculated by dividing the number of days between oviposition and the appearance of a specific character by the number of days between oviposition and egg hatch.

## Results and Discussion

### Oviposition behaviors

The mean ± SE total time (from ovipositor insertion to withdrawal, Videos 1 and 2) for a successful oviposition in the agarose substrate by a mated female *L. hesperus* was 31 ± 1.5 sec. Of the total time required for oviposition in the agarose, a mean of 11 ± 1.0 sec was required for insertion of the ovipositor, 17 ± 0.9 sec for deposition of the egg, and 3 ± 0.2 sec for withdrawal of the ovipositor. The act of depositing the egg was accompanied by abdominal contractions and could be subdivided into passage of the egg along the medial oviduct to the gonopore (9 ± 1.4 sec; beginning with the initiation of muscle contractions and ending with emergence of the egg between the lateral halves of the ovipositor) and passage of the egg along the length of the ovipositor (8 ± 1.3 sec). In comparison with agarose, the mean total time for oviposition in green bean pods was 43 ± 2.0 sec. The longer and more variable time for oviposition in green bean pods suggests the total time required for oviposition may vary among different oviposition substrates.

About 44% of oviposition attempts in agarose and 59% of oviposition attempts in green bean pods were not successful. During unsuccessful oviposition attempts, the ovipositor was inserted into the substrate, but abdominal contractions typical of those exhibited during successful oviposition were not observed. Both the time required for oviposition and the proportion of successful attempts may represent parameters of interest in efforts to characterize host plant tolerance or nonpreference to *L. hesperus*.

**Video 1.**
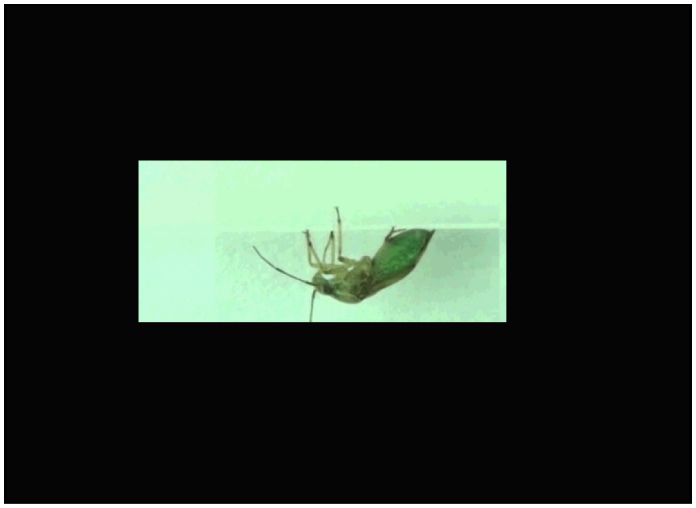
Lygus *hesperus* oviposition event with white background to facilitate the view of the paths of the stylets and ovipositor. Click image to view video. Download Video

**Video 2.**
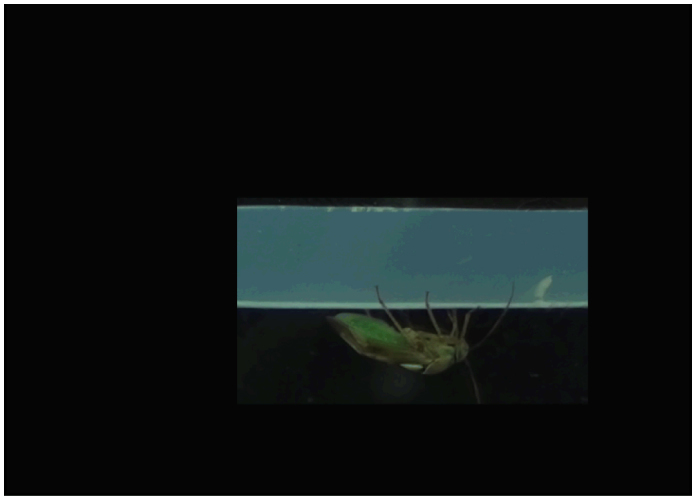
Lygus *hesperus* oviposition event with black background to facilitate view of the passage of the egg from the gonopore down the length of the ovipositor. Click image to view video. Download Video

Each female stylet-probed the substrate before inserting the ovipositor, regardless of oviposition substrate or whether oviposition was successful (Video 1). The association of stylet probing with oviposition was previously reported for *Lygus* spp. (Romani et al. 2005; Cooper and Spurgeon 2011) and for mirids of other genera (Ferran et al. 1996; Wheeler 2001). The mean ± SE total duration of styletprobes associated with oviposition in agarose was 8 ± 1.1 sec, whereas the duration of probing on green bean pods averaged 9 ± 1.2 sec. These times were consistent with the duration of ovipositional stylet-probes previously observed on cotton floral buds (Cooper and Spurgeon 2011). Observations of oviposition in agarose indicated the path of ovipositor insertion was closely associated with the paths and depths of the stylet-probes (Video 1).

**Figure 1.**
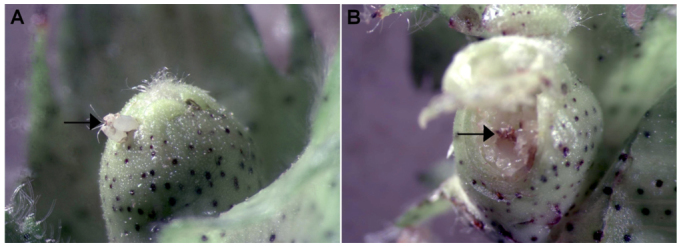
(A) Field collected cotton (Phytogen 72) floral bud with two *Lygus* spp. eggs oviposited through the bracts (bracts were removed to view the eggs) in the region of the square containing the anthers. (B) Partial dissection of the floral bud to reveal injury to the developing anthers at the location of oviposition. High quality figures are available online.

Several potential roles for the ovipositional stylet-probes exhibited by mirids have been proposed, including locating, marking, and softening oviposition sites (Ferran et al. 1996; Boyd et al. 2002; Boyd 2003; Romani et al. 2005; Salerno et al. 2007). Whereas feeding probes are associated with injection of digestive enzymes, including pectinases (i.e., polygalacturonases), proteases, and amylases (Strong and Kruitwagen 1968; Agusti and Cohen 2000; Zeng and Cohen 2000; Celorio-Mancera et al. 2008; Celorio-Mancera et al. 2009), it is not known whether *Lygus* spp. inject saliva while stylet-probing oviposition sites. We have observed eggs deposited in the developing anthers of floral buds collected from field grown cotton (unpublished data). In each of these instances, anthers surrounding the egg exhibited injury that was identical to the injury caused by *L. hesperus* feeding (Figure 1). Visible injury to floral buds caused by mirid feeding is induced by the enzymatic degradation of plant tissues and not by mechanical damage (Strong 1970; Shackel et al. 2005). These observations suggest that *L. hesperus* inject saliva during stylet-probes associated with oviposition. Therefore, at least observational evidence of saliva injection during the ovipositional probes of *L. hesperus* exists.

Eggs embedded into plant tissues are subject to mechanical pressures associated with plant growth (Southwood 1956; Hartley 1965). Furthermore, eggs of *Lygus* spp. expand as the embryos develop (Stewart and Gaylor 1993; this study), which may impose additional mechanical pressures on the eggs. The digestive enzymes in saliva injected during ovipositional stylet-probes could play a role of diminishing mechanical pressures on the eggs by weakening or degrading plant tissues surrounding the eggs.

Plant proteins that inhibit the activity of polygalacturonase have been identified (Frati et al. 2006). Within certain crop species, variability in the activity of polygalacturonase inhibiting enzymes is related to the extent of injury caused by *Lygus* feeding (Shackel et al. 2005). If salivary polygalacturonases soften plant tissues surrounding eggs and this role is important for egg survival, then selection for plants producing high levels of polygalacturonase inhibiting enzymes may represent a useful source of antibiosis. The potential role of ovipositional stylet-probes and associated salivary enzymes in determining survival of *L. hesperus* eggs warrants further research.

### Observations of embryo development

**Figure 2.**
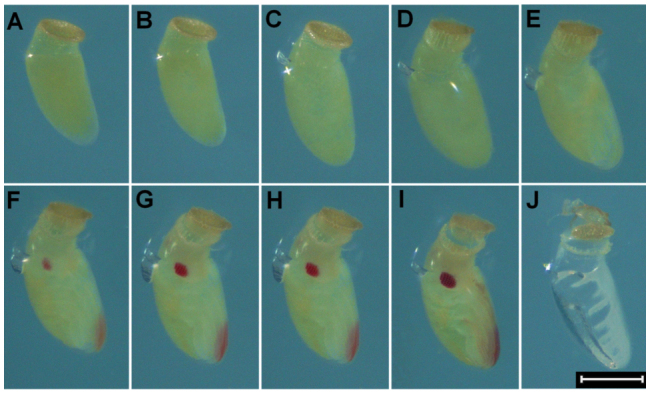
Right lateral view of Lygus *hesperus* embryonic development. (A) < 6 hours after oviposition, (B) 1 day after oviposition, (C) 2 days after oviposition, (D) 3 days after oviposition, (E) 4 days after oviposition, (F) 5 days after oviposition, (G) 6 days after oviposition, (H) 7 days after oviposition, (I) 8 days after oviposition, and (J) 9 days after oviposition. The measurement bar represents 300 µm. High quality figures are available online.

**Figure 3.**
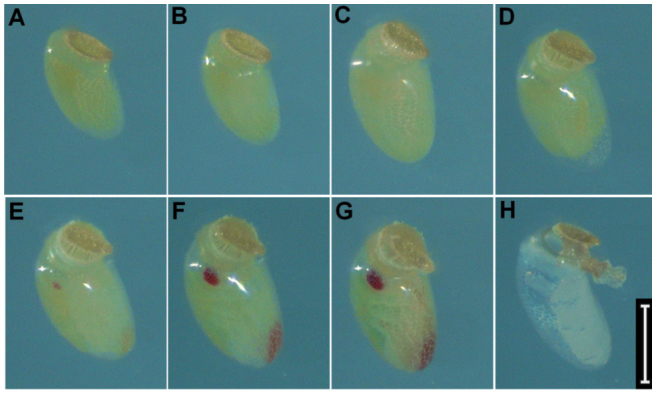
Right lateral/ventral view of *Lygus hesperus* embryo development. (A) < 6 hours after oviposition, (B) 1 day after oviposition, (C) 2 days after oviposition, (D) 3 days after oviposition, (E) 4 days after oviposition, (F) 5 days after oviposition, (G) 6 days after oviposition, and (H) 7 days after oviposition. The measurement bar represents 300 µm. High quality figures are available online.

**Figure 4.**
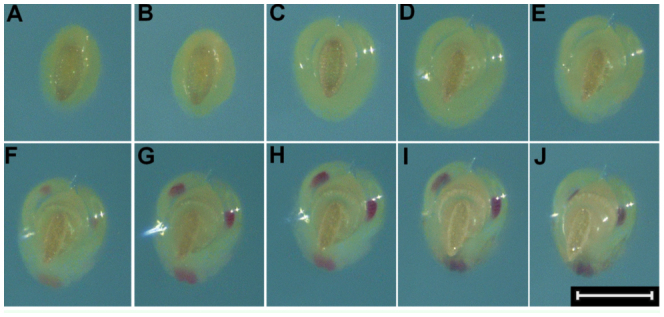
Anterior view of *Lygus hesperus* embryo development. (A) < 6 hours after oviposition, (B) 1 day after oviposition, (C) 2 days after oviposition, (D) 3 days after oviposition, (E) 4 days after oviposition, (F) 5 days after oviposition, (G) 6 days after oviposition, (H) 7 days after oviposition, (I) 8 days after oviposition, and (J) 9 days after oviposition. The measurement bar represents 300 µm. High quality figures are available online.

Newly oviposited eggs of *L. hesperus* averaged about 250 µm in width (longest dimension of a roughly oval cross-section) and 700 pm in length (measured between the anterior and posterior poles) (Figures 2A, 3A, and 4A). These dimensions are slightly smaller than those of newly oviposited eggs of *L. lineolaris*, which are about 270–300 µm in width and 950–1000 µm in length (Stewart and Gaylor 1993; Ma et al. 2002). The mean ± SE egg development time in agarose at 26.6 °C was 8.9 ± 0.33 days.

At 26.6 °C, little change in the appearance of *L. hesperus* eggs occurred during the first 24 hours following oviposition (Figures 2B, 3B, and 4B). Egg size subsequently increased to approximately 320 µm in width and > 800 µm in length at 2.3 ± 0.14 days (0.3 ± 0.02 of total egg development time) (Figures 2C, 3C, and 4C). After this initial increase, egg size remained relatively constant through the remainder of the developmental period (Figures 2, 3, and 4). Based on reports of other Hemiptera with endophytic eggs (Hartley 1965; Constant et al. 1994), the observed increase in egg size was probably associated with water uptake from surrounding plant tissue. The observed egg swelling was also consistent with reports of *L. lineolaris* eggs (Stewart and Gaylor 1993). Eggs from unmated *L. hesperus* females did not exhibit swelling, suggesting that this increase in size is associated with presence of a viable embryo.

At 3.5 ± 0.21 days after oviposition (0.4 ± 0.02 of total development time), the operculum became extended and separated from the chorion (Figures 2D, 3D, and 4D), creating a gap that was bridged by the serosal cuticle. This extension of the operculum can usually be observed for eggs deposited into plant tissues. Extension of the operculum also occurs during development of *L. lineolaris* eggs (Stewart and Gaylor 1993). As for egg swelling, extension of the operculum was not observed for unfertilized eggs, indicating this condition is indicative of the presence of a developing embryo.

Katatrepsis, or migration of the embryo to position the head at the anterior pole of the egg (Dorn 1976), was indicated by the appearance of empty space between the embryo and chorion near the posterior of the egg (Figure 3D). Katatrepsis was observed in conjunction with extension of the operculum (3.7 ± 0.17 days after oviposition, 0.4 ± 0.02 of total development time). The timing of katatrepsis observed in *L. hesperus* eggs was roughly similar to that reported for eggs of *Oncopeltus fasciatus* (Dorn 1976).

**Figure 5.**
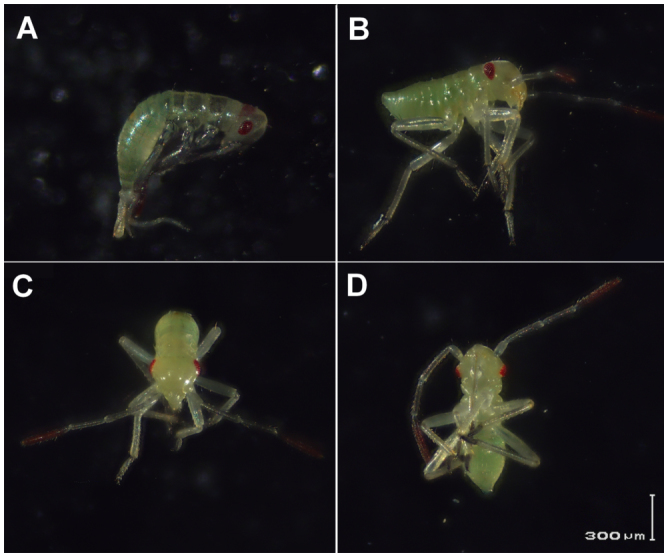
Lygus *hesperus* 1^st^ instar nymph. (A) Right lateral view < 1 hour after eclosion, (B) right lateral view < 24 hours after eclosion, (C) anterior view < 24 hours after eclosion, and (C) ventral view < 24 hours after eclosion. High quality figures are available online.

Visual evidence of tagmosis and the presence of appendages were observed 4.1 ± 0.14 days after oviposition (0.5 ± 0.02 of total development time; Figures 2E and 3E). Photographs of newly emerged 1^st^ instars are provided as reference for the proportions of body segments and positions of appendages (Figure 5). Red pigmentation of the distal antennal segments and eyes was first observed 5.0 ± 0.15 days and 5.3 ± 0.18 days after ovipostion (0.6 ± 0.02 of total development time), respectively (Figure 2F, 3E, and 4F). Stewart and Gaylor (1993) reported that in *L. lineolaris* embryos red pigmentation appeared in the eyes before it appeared on the antennae, which is inconsistent with our observations of *L. hesperus* embryos. Once visible, the pigmentation gradually intensified until the eggs hatched (Figures 2, 3, and 4).

Legs, antennae, and the proboscis darkened and became more distinctly visible 7.6 ± 0.27 days after oviposition (0.9 ± 0.01 of the total development time; Figures 2I, 3G, and 4I). This darkening likely coincided with apolysis of the second embryonic cuticle and deposition of the third embryonic cuticle, which in the embryo of *O. fasciatus* also occurs at about 0.8 of total development (Dorn 1976). Ecdysis of the second embryonic cuticle does not occur until hatch, and the third embryonic cuticle is the cuticle of the newly-hatched first instar (Dorn 1976).

The embryo migrated toward the anterior pole such that the red eyespots were visible through the serosal cuticle connecting the chorion and the operculum 7.5 ± 0.27 days after oviposition (0.9 ± `0.01 of total development time; Figure 4J). The appearance of the red eyes above the surface of the oviposition substrate was indicative of imminent egg hatch. After hatch, the chorion appeared nearly transparent, the operculum was free of the chorion through most of its circumference, and the whitish remnants of the second embryonic cuticle were visible through the chorion (Figures 2J and 3H).

Studies of insect development are often difficult to conduct or interpret at temperatures near the lower or upper thresholds. In the case of *L. hesperus*, which deposit their eggs in the host plant, studies near the lower developmental threshold are particularly difficult because the oviposition substrate may deteriorate and destroy the eggs before embryonic development is complete (WR Cooper, unpublished data). Even when using a non-living substrate such as agarose, the period of egg development may be so extended that failure to hatch within a specified time is interpreted as failure to develop. The ability to discern phases of embryonic development will allow the design of experiments to better understand the lowtemperature ecology of this species. In addition, positive identification of characteristics of egg development that are (1) consistent indicators of egg fertility and (2) observable when eggs are deposited within opaque plant material should have practical utility in studies of mating success and fecundity. Finally, recognition that appearance of the embryo with red eyespots above the surface of the oviposition substrate is an indication of imminent hatch facilitates scheduling of activities in laboratory experiments where large numbers of newlyeclosed nymphs are needed. Extension of the operculum and appearance of the redpigmented eyes may also be useful in assessing egg population age structure from field censuses.
